# Supplementary material for: Socioeconomic and contextual correlates of suicidal ideation among Indonesian adults: Evidence from a multilevel analysis of the 2018 National Health Survey
Source: PLoS One. 2026 Apr 15;21(4):e0344394. doi: 10.1371/journal.pone.0344394 (PMC13082633; doi:10.1371/journal.pone.0344394)
Supplement: S1 Table — (DOCX) [file pone.0344394.s001.docx]

**S1 Table. Characteristics of participants**

| **Variables** | **N** | **% or mean** | **SD** | **Min** | **Max** |
| --- | --- | --- | --- | --- | --- |
| Individual variables |  |  |  |  |  |
| Suicidal ideation | 5,507 | 0.9% |  | 0 | 100 |
| Sex |  |  |  |  |  |
| Male | 334,368 | 52.6% |  | 0 | 100 |
| Female | 301,917 | 47.4% |  | 0 | 100 |
| Marital status |  |  |  |  |  |
| Never married | 97,023 | 15.2% |  | 0 | 100 |
| Married | 478,102 | 75.1% |  | 0 | 100 |
| Divorced | 13,943 | 2.2% |  | 0 | 100 |
| Widowed | 47,217 | 7.4% |  | 0 | 100 |
| Age group |  |  |  |  |  |
| Age (Mean) |  | 42.30 | 15.2 | 18 | 97 |
| 18-24 | 86,202 | 13.5% |  | 0 | 100 |
| 25-34 | 131,765 | 20.7% |  | 0 | 100 |
| 35-44 | 151,070 | 23.7% |  | 0 | 100 |
| 45-54 | 127,348 | 20.0% |  | 0 | 100 |
| 55-64 | 84,443 | 13.3% |  | 0 | 100 |
| 65-74 | 38,487 | 6.0% |  | 0 | 100 |
| >=75 | 16,970 | 2.7% |  | 0 | 100 |
| Education |  |  |  |  |  |
| Never go to school | 40,755 | 6.4% |  | 0 | 100 |
| Less than primary school | 85,184 | 13.4% |  | 0 | 100 |
| Primary school | 159,119 | 25.0% |  | 0 | 100 |
| Junior high school | 112,008 | 17.6% |  | 0 | 100 |
| Senior high school | 177,651 | 27.9% |  | 0 | 100 |
| Diploma | 20,223 | 3.2% |  | 0 | 100 |
| College/University | 41,345 | 6.5% |  | 0 | 100 |
| Employment status |  |  |  |  |  |
| Unemployed | 181,711 | 28.6% |  | 0 | 100 |
| Students | 18,694 | 2.9% |  | 0 | 100 |
| Civil servant/army/policeman | 27,429 | 4.3% |  | 0 | 100 |
| Private worker | 50,779 | 8.0% |  | 0 | 100 |
| Self-employed | 91,160 | 14.3% |  | 0 | 100 |
| Farmer | 16,2501 | 25.5% |  | 0 | 100 |
| Fisherman | 82,99 | 1.3% |  | 0 | 100 |
| Driver/household assistance | 52,802 | 8.3% |  | 0 | 100 |
| Others | 42,910 | 6.7% |  | 0 | 100 |
| Joint diseases |  |  |  |  |  |
| No | 582,279 | 91.5% |  | 0 | 100 |
| Yes | 54,006 | 8.5% |  | 0 | 100 |
| Hypertension |  |  |  |  |  |
| No | 580,428 | 91.2% |  | 0 | 100 |
| Yes | 55,857 | 8.8% |  | 0 | 100 |
| Stroke |  |  |  |  |  |
| No | 629,353 | 98.9% |  | 0 | 100 |
| Yes | 6,932 | 1.1% |  | 0 | 100 |
| Diabetes |  |  |  |  |  |
| No | 622,098 | 97.8% |  | 0 | 100 |
| Yes | 14,187 | 2.2% |  | 0 | 100 |
| Heart diseases |  |  |  |  |  |
| No | 624,058 | 98.1% |  | 0 | 100 |
| Yes | 12,227 | 1.9% |  | 0 | 100 |
| Asthma |  |  |  |  |  |
| No | 619,255 | 97.3% |  | 0 | 100 |
| Yes | 17,030 | 2.7% |  | 0 | 100 |
| Cancer |  |  |  |  |  |
| No | 634,575 | 99.7% |  | 0 | 100 |
| Yes | 1,710 | 0.3% |  | 0 | 100 |
| Renal failure |  |  |  |  |  |
| No | 633,665 | 99.6% |  | 0 | 100 |
| Yes | 2,620 | 0.4% |  | 0 | 100 |
| Smoking status |  |  |  |  |  |
| Everyday | 172,394 | 27.1% |  | 0 | 100 |
| Not everyday | 29,655 | 4.7% |  | 0 | 100 |
| Ex smoker | 33,161 | 5.2% |  | 0 | 100 |
| Not smoker | 401,075 | 63.0% |  | 0 | 100 |
| Alcohol consumption |  |  |  |  |  |
| Under standard | 17,787 | 2.8% |  | 0 | 100 |
| More than standard | 12,643 | 2.0% |  | 0 | 100 |
| No alcohol | 605,855 | 95.2% |  | 0 | 100 |
| Self-rated health |  |  |  |  |  |
| Good | 450,805 | 70.8% |  | 0 | 100 |
| Adequate | 172,535 | 27.1% |  | 0 | 100 |
| Poor | 12,945 | 2.0% |  | 0 | 100 |
| Household expenditure |  |  |  |  |  |
| 1st quartile | 112,681 | 17.7% |  | 0 | 100 |
| 2nd | 119,954 | 18.9% |  | 0 | 100 |
| 3rd | 126,206 | 19.8% |  | 0 | 100 |
| 4th | 133,751 | 21.0% |  | 0 | 100 |
| 5th | 143,693 | 22.6% |  | 0 | 100 |
| Island |  |  |  |  |  |
| Outer Java | 424,298 | 66.7% |  | 0 | 100 |
| Java | 211,987 | 33.3% |  | 0 | 100 |
| District/community variables |  |  |  |  |  |
| Community social capital |  | 0.14 | 1.05 | -1.34 | 5.68 |
| Social deprivation |  | 0.13 | 1.07 | -1.06 | 6.65 |
| District GDP |  | 9.43 | 1.23 | 4.99 | 1003.01 |
